# The Antioxidant and Antibacterial Properties of Honeycomb-Structured Polylactic Acid/Astaxanthin@ZIF-8 Bio-Composite Film Applied in Beef Preservation

**DOI:** 10.3390/biom16081184

**Published:** 2026-08-13

**Authors:** Sheng Liu, Shuran Xing, Feifei Wang, He Zhu, Xiaoyun Fu, Yang Yu, Litao Wang

**Affiliations:** 1 College of Food Science and Engineering, Shandong Agriculture and Engineering University, No. 866, Nongganyuan Road, Licheng District, Jinan 250100, China; z2023066@sdaeu.edu.cn (S.L.); xingshuran12138@gmail.com (S.X.); feiake880818@gmail.com (F.W.); z2013428@sdaeu.edu.cn (H.Z.); z2017025@sdaeu.edu.cn (Y.Y.); 2 School of Pharmacy, Jining Medical University, Jining 272000, China; mikeggrb@gmail.com

**Keywords:** honeycomb-structured, polylactic acid/AST@ZIF-8, antibacterial, antioxidant

## Abstract

Food spoilage is mainly driven by lipid oxidation and microbial proliferation. To counteract these challenges, multifunctional protective films have emerged as effective solutions for suppressing both reactions. In this work, biodegradable polylactic acid (PLA) was selected as the polymer matrix to fabricate composite films. Colloidal ZIF-8 nanoparticles loaded with the natural antioxidant astaxanthin (AST) were mixed with PLA. By precisely controlling temperature and humidity during the solution-casting process, a honeycomb-structured PLA/AST@ZIF-8 composite film was successfully prepared. The structural and physico-chemical properties of the as-prepared film were systematically characterized by FE-SEM, FTIR, Raman, XRD, and TGA. The results confirm the formation of a well-defined honeycomb morphology, with AST uniformly dispersed throughout the PLA matrix. Importantly, films with AST content above 2.0% exhibit weak antibacterial properties. Practical application tests for preserving beef tenderloin demonstrate that the film provides excellent antioxidant and antibacterial effects. On the seventh day, the water loss, pH, *ΔE* and total volatile basic nitrogen (TVB-N) values of beef samples treated with the PLA/ZIF-8/2.5%AST film remained within acceptable thresholds, effectively extending the shelf life of fresh meat. Safety assessments on overall migration (OM) and Zn^2+^-specific migration confirm that the PLA/AST@ZIF-8 composite film fully complies with EU Regulation No. 10/2011. These results demonstrate the great application prospects of the composite film for high-performance food preservation.

## 1. Introduction

Food spoilage stands as a pressing global challenge within the entire food industry chain. As a complex biological process, it arises from the combined effects of multiple factors, including microbial proliferation, enzymatic reactions, and lipid oxidation [1,2,3,4]. In addition, mitigating food spoilage can reduce agricultural ammonia emissions, relieve PM2.5-related pollution, and ease ecosystem damage induced by nitrogen deposition [5]. Therefore, developing safe, efficient, and environmentally friendly technologies is of great significance for maintaining food freshness and addressing spoilage [6,7]. Traditional preservation strategies mainly depend on low-temperature storage (4–8 °C), modified-atmosphere packaging (MAP, typically with oxygen content below 0.5%), and chemical preservatives such as potassium sorbate. Nevertheless, these approaches suffer from inherent drawbacks, including insufficient shelf life improvement, high-energy consumption, and risks associated with chemical preservative residues. In recent years, bioactive biodegradable packaging has attracted much attention due to its combination of physical barriers and chemical antibacterial functions [8], and is regarded as one of the most promising packaging alternatives in the post-plastic era. For instance, biodegradable PBAT/PBS nanocomposite films reinforced with zinc oxide were prepared by cast extrusion; these films exhibited improved antibacterial efficacy and delayed the discoloration of red pigments in packaged minced pork [9]. Fang et al. prepared the composite edible films of chitosan with essential oil and the films were applied to ready-to-eat fish balls antimicrobial, which reduced total bacterial counts and total volatile basic nitrogen during 12 days storage [10]. Huang et al. developed carboxymethyl cellulose nanofibers grafted with enzyme-like metal–organic frameworks (MOFs) bioinspired spray coating for banana and mango preservation, which enabled effective enzyme-like sterilization against three typical foodborne pathogens with killing rates as high as 90.46% [11].

Polylactic acid (PLA), a renewable and biodegradable polymer, exhibits favorable biocompatibility, outstanding film-forming capacity, and desirable mechanical strength [12,13]. Nevertheless, the intrinsic hydrophobic nature of PLA leads to low loading efficiency for bioactive molecules, and its rigid molecular-chain configuration readily triggers brittle fracture of resultant films [14]. To address these limitations, electrospun nanofibers have been explored as an effective strategy to endow PLA matrices with improved loading capacity for bioactive substances while tuning mechanical performance. Spinning techniques enable the fabrication of nanofibers with distinctive core–shell architectures. Featuring small diameters, large specific surface areas and high porosity, such nanofibers represent ideal carriers for encapsulating active components [15]. Han et al. fabricated the PLA/cinnamaldehyde/tea polyphenol coaxial antibacterial nanofibers by coaxial electrospinning technology and the prepared composite materials had strong hydrophobicity, good thermal stability and mechanical properties [16]. Li et al. prepared antibacterial electrospun PLA/ZnO composite films for chicken meat preservation, and the preservation experiments revealed that the above films (PLA/ZnO-1.0%, PLA/ZnO-0.5%) extended the cold storage period of chicken meat by 3 days [17]. Although PLA composite films have demonstrated promising applications in food preservation owing to their superior structural properties, the fabrication of PLA nanofibers typically demands specialized equipment and stringent processing conditions, posing a significant barrier to their large-scale commercialization. Therefore, the development of a simple, scalable, and multifunctional PLA composite film preparation technology is crucial for enhancing food safety and quality.

Apart from electrospinning-derived nanostructures, incorporating functional nanofillers into polymer matrices offers another feasible route to construct high-performance biodegradable packaging films. Some nanomaterials (such as ZnO, TiO_2_, MOFs, etc.) have been studied for their improvement in mechanical, barrier, thermal and antimicrobial properties when incorporated into the polymer matrices [18]. Mousavi et al. demonstrated that natural bioactive compounds sinapic acid and apigenin were encapsulated within zeolitic imidazolate frameworks-8 (ZIF-8) and ZIF-67 frameworks to achieve pH-responsive controlled release and enhanced antibacterial performance [19]. Nanomaterial incorporation has been widely investigated to improve the functional performance of polymer films without sacrificing processability, highlighting the critical role of microstructure modulation in performance enhancement [9]. Therefore, regulating the microstructural architecture of PLA via nanoparticle integration provides a feasible strategy to strengthen the adsorption capacity and comprehensive performance of PLA-based films. In recent years, MOFs have emerged as ideal carriers for loading active molecules due to their unique porous structure. Such unique porous architecture enables MOFs to act as efficient delivery platforms for natural bioactive substances across food-related applications. MOFs have been successfully applied in multiple food-industry fields, including impurity adsorption, active food packaging fabrication, and food quality monitoring [20]. Benefiting from excellent biocompatibility, MOFs have been increasingly recognized as reliable functional additives for high-performance food packaging systems [21,22,23,24]. The incorporation of MOFs into polymer matrices not only improves the water and oxygen barrier properties as well as mechanical robustness of composite films but also integrates additional favorable functionalities, such as light-shielding capability, microstructure optimization, and antibacterial activity [25,26]. Among various MOF derivatives, ZIF-8 possesses a large specific surface area, high porosity, tunable pore structure, and superior loading capability, making it one of the most promising nanofillers for constructing multifunctional inorganic–polymer hybrid food packaging materials [27,28,29]. With unique porous structure and molecular immobilization properties, ZIF-8 can efficiently load and stabilize natural bioactive molecules, significantly improving the sustained preservation performance of PLA composite films.

Astaxanthin (AST), a natural lipid-soluble bioactive molecule recovered from shrimp shell waste, exhibits superior antioxidant and broad-spectrum antibacterial bioactivities, rendering it a promising biomolecular preservative for food safety improvement and shelf life extension [30,31]. Numerous biomolecular studies have confirmed that AST effectively scavenges reactive oxygen species (ROS) and disrupts bacterial metabolic activity, which endows it excellent preservation potential in food systems [32,33,34]. Benefiting from these inherent biological properties, AST has been widely applied as a natural antioxidant, colorant, and biopreservative in food industrial practice [35]. For example, Mussagy et al. demonstrated that AST-mediated microbial inhibition and antioxidant protection significantly delayed strawberry deterioration and prolonged the postharvest shelf life [36]. Nevertheless, the practical application of pure AST is severely restricted by its low structural stability, poor aqueous dispersibility, and susceptibility to oxidative degradation under ambient conditions. Encapsulation using porous nanocarriers has been verified as an efficient biomolecular modification strategy to stabilize and controllably deliver bioactive compounds [37]. Owing to its unique porous structure, ZIF-8 can efficiently anchor and encapsulate AST molecules, thereby enhancing the structural stability of AST and retaining its intrinsic antioxidant and antibacterial bioactivities.

This work reports the fabrication of high-performance PLA/AST@ZIF-8 composite films for active food preservation. With ZIF-8 serving as both an effective AST carrier and a microstructure modifier, regular honeycomb porous structures were formed in PLA films under precisely regulated preparation conditions. The structural and antibacterial properties of the composite films were characterized in detail. Fresh beef tenderloin was used as a food model to evaluate the practical preservation effect. The results indicated that the developed composite films could efficiently retard the deterioration of fresh beef and remarkably extend its shelf life during cold storage.

## 2. Experimental Section

### 2.1. Reagents and Materials

2-methylimidazole, PLA and methanol were purchased from Shanghai Maclin Biochemical Technology Co., Ltd (Shanghai, China). Zn(NO_3_)_2_⋅6H_2_O was purchased from China National Pharmaceutical Group Chemical Reagent Co., Ltd. (Shanghai, China). Trichloromethane (CHCl_3_, analytical grade) was purchased from Yantai Far East Fine Chemical Co., Ltd (Yantai, China). AST was purchased from Nanjing Dulai Biotechnology Co., Ltd (Nanjing, China). A total of 38 kg of beef tenderloin was purchased at the local agricultural market (Zibo, China). Ultrapure water (>18 MΩ·cm) was used throughout the experiment.

### 2.2. Preparation of PLA/AST@ZIF-8 Composite Materials

#### 2.2.1. Preparation of ZIF-8

Firstly, 1.50 g of Zn(NO_3_)_2_⋅6H_2_O and 3.32 g of 2-methylimidazole were dissolved in 150 mL of methanol, stirred at 35 °C for 18 h, centrifuged, and washed 2–3 times with methanol. The reaction product ZIF-8 was obtained by vacuum drying at 70 °C for 18–24 h.

#### 2.2.2. Preparation of PLA/ZIF-8 Composite Material

Next, 5.00 g PLA was dissolved in 100 mL of CHCl_3_ and stirred magnetically in a water bath at 50 °C for 0.5 h until the solution became transparent, and then 0.050 g of ZIF-8 (1.0 wt% PLA) was added and stirred magnetically overnight. Using the solution casting method, 10 mL of the above solution was poured into a culture dish, and at 35 °C and 80% humidity, the PLA/ZIF-8 composite film was obtained overnight using a constant temperature and humidity incubator (YinggongHWS-50B, Xuchang, China).

#### 2.2.3. Preparation of PLA/AST@ZIF-8 Composite Materials

**Figure 1:**
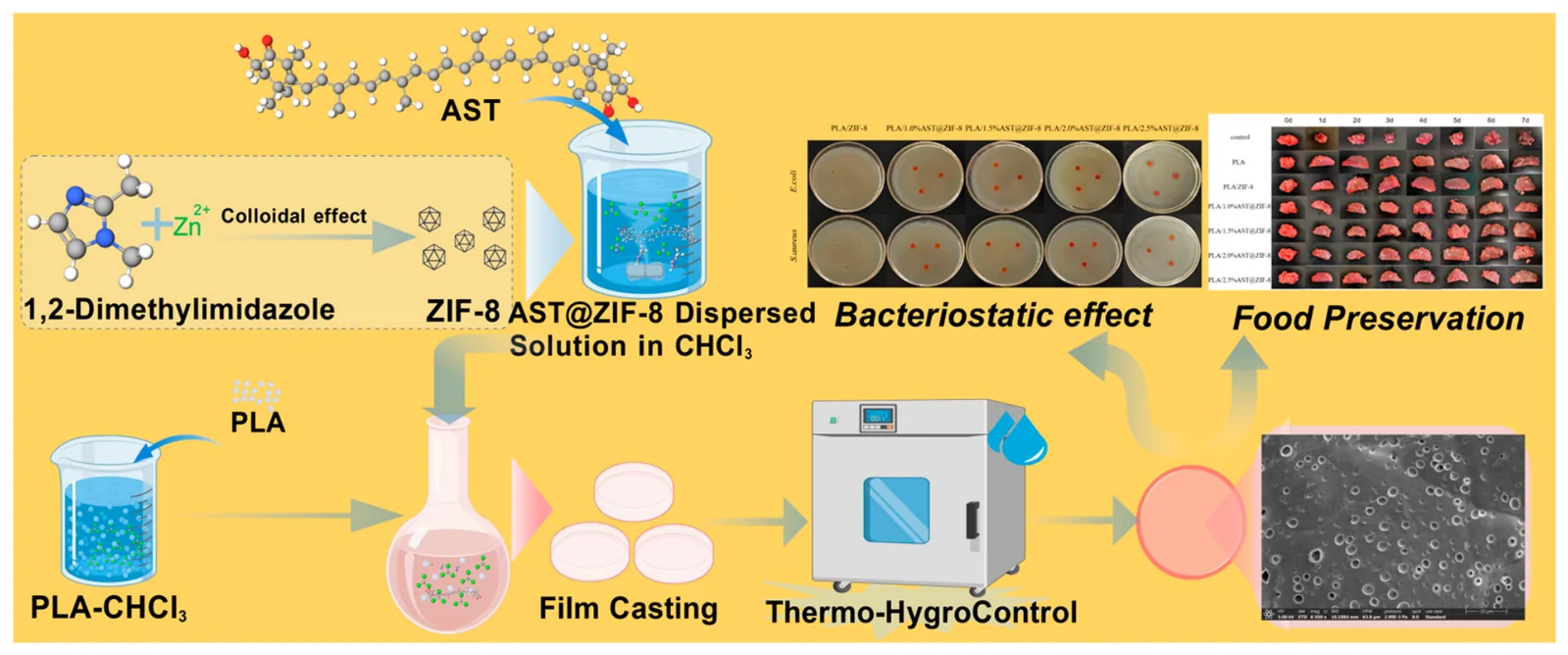
Illustration of the procedure used for the fabrication of PLA/AST@ZIF-8 applied to beef tenderloin preservation packaging.

AST (1.0, 1.5, 2.0, 2.5 wt% PLA) and 0.050 g ZIF-8 (1.0 wt% PLA) were separately weighed and added into a conical flask, and then 100 mL CHCl_3_ was added and stirred magnetically for 12 h until there were no obvious AST particles. The preparation of PLA solution was carried out according to 2.2.2. The weighed AST and ZIF-8 were added to the PLA solution, and the mixed solution was stirred for another 120 min. The composite films were prepared following the procedure described in Section 2.2.2, as illustrated in Figure 1.

### 2.3. Structure and Performance Characterizations of Composite Films

#### 2.3.1. Characterization of Sample Structure

A vortex mixer (Vortex-Genie 2, Scientific Industries, Bohemia, NY, USA ) and refrigerated centrifuge (A16K-S, Anjunyan, Changsha, China) were used to pretreat samples. The morphology of the resulting products was observed by a FE-SEM (FEI Apreo SEM, Thermo Fisher Scientific, Hillsboro, OR, USA). The material structure characterization was completed by X-ray diffraction (XRD, Rigaku, MiniFlex600, Akishima, Tokyo, Japan) patterns at scan angle of 10–90° and a scan speed of 10°/min, FTIR (Nicolet iS5, Thermo Fisher Scientific, Waltham, MA, USA ) and Raman spectrometer (LabRAM HR evolution, HORIBA Scientific, Longjumeau, France). Thermal stability analysis was done using the thermogravimetric analyzer (TGA, Netzsch STA449C, NETZSC-Gerätebau GmbH, Selb, Bavaria, Germany ). The colorimeter (SC-10, Shenzhen Sanenshi Technology, Shenzhen, China), pH meter (testo205, German Instrument (Shenzhen), Shenzhen, China), Electronic Balance (JE3001, Shanghai Puchun Measuring Instrument, Shanghai, China) were used to measure the color, pH and weight loss of samples. A contact angle tester (DSA20, KRÜSS GmbH, Hamburg, Germany) and oxygen permeability (OP) tester (C103M, Labthink, Jinan, China), ICP-MS (Agilent, 7850, Agilent Technologies, Santa Clara, CA, USA) were employed to measure the Zn^2+^ content in the migration solution.

#### 2.3.2. Antibacterial Activity of the Composite Films

Firstly, take two 5 mL LB liquid culture media and inoculate them with *Staphylococcus aureus* (*S. aureus*) and *Escherichia coli* (*E. coli*), respectively, and then shake and culture at 37 °C and 220 r/min for 18 h. Secondly, prepare two bottles of 500 mL LB solid culture media and sterilize under high pressure, cool down, and add 2.5 mL of bacterial solution to each bottle. Shake well and pour onto a plate. Use a 0.6 cm puncher to punch holes into a PLA/ZIF-8 film and four different samples of PLA/AST@ZIF-8 film to prepare bacterial slides. Stick the bacterial slide onto the surface of the agar inoculated with the test bacteria. Observe and measure the size of the inhibition zone after 24 h of cultivation at 37 °C.

#### 2.3.3. Weight Loss

Weigh an appropriate amount of beef sample, film it, record its initial mass as *W_1_*, measure and record the sample mass according to different storage times, and record it as *W_2_*. Three parallel samples are set for each group, and the weight loss rate calculation formula is as follows:

Weight loss (%) = (*W_1_* − *W_2_*)/*W_1_* × 100 (1)

#### 2.3.4. pH

Weigh 5 g of beef samples and use a pH meter to measure the pH values of beef samples stored for different durations. Before measuring, calibrate the pH electrode with a buffer solution with a pH value of 7.00 or 4.00. Insert the pH electrode into the center of the beef sample during measurement and prepare three parallel samples for each group. Repeat the experiment three times.

#### 2.3.5. Color

The color parameters of beef samples were determined using the method proposed by Ruan et al. [38]. The colorimeter was used to measure the L* (brightness/darkness), a* (redness/greenness), and b* (yellowness/blurriness) values of the sample. At least 5 different sites should be selected for each sample to complete the detection, and the experiment should be independently repeated at least 3 times. The calculation formula for total color difference (△E*) is as follows:

(2) $${\Delta E}^{*}={\;[{\left({\Delta L}^{*}\right)}^{2}+{\left({\Delta a}^{*}\right)}^{2}+{\left({\Delta b}^{*}\right)}^{2}]}^{1/2}$$

#### 2.3.6. Determination of Total Volatile Basic Nitrogen (TVB-N)

TVB-N of beef was detected according to Chinese Standard (GB 5009.228-2016) at different storage times [39]. Weigh 10 g of meat sample, add 75 mL of water and mix well. Place it in a distillation tube and soak for 30 min for later use. Firstly, perform a reagent blank measurement and record the blank value. Add 1 g of MgO to the distillation tube containing the processed sample, add 10 mL of boric acid and 5 drops of mixed indicator to the receiving bottle, quickly connect to the distillation apparatus for measurement, and take 3 parallel samples for each measurement. Repeat the measurement 3 times for each sample and take the average value.

### 2.4. Safety Evaluation of Prepared Films

The total migration from the film into aqueous food simulants is tested according to GB 5009.156-2016 [40]. Herein, 4.0 *w*/*v*% acetic acid water solution and 10%, 20%, 50% and 65% (*v*/*v*) ethanol water solutions were used as aqueous food simulants. Zn^2+^ migration detection was directly performed on the 4.0 *w*/*v*% acetic acid leaching solution.

### 2.5. Statistical Analysis

All data were expressed as mean ± standard deviation (SD). When *p* < 0.05, the differences were statistically significant. Statistical analysis was performed using Origin 2021 and Excel 2021 software.

## 3. Results and Discussion

### 3.1. Microstructure Characterizations of PLA, PLA/ZIF-8 and PLA/AST@ZIF-8

**Figure 2:**
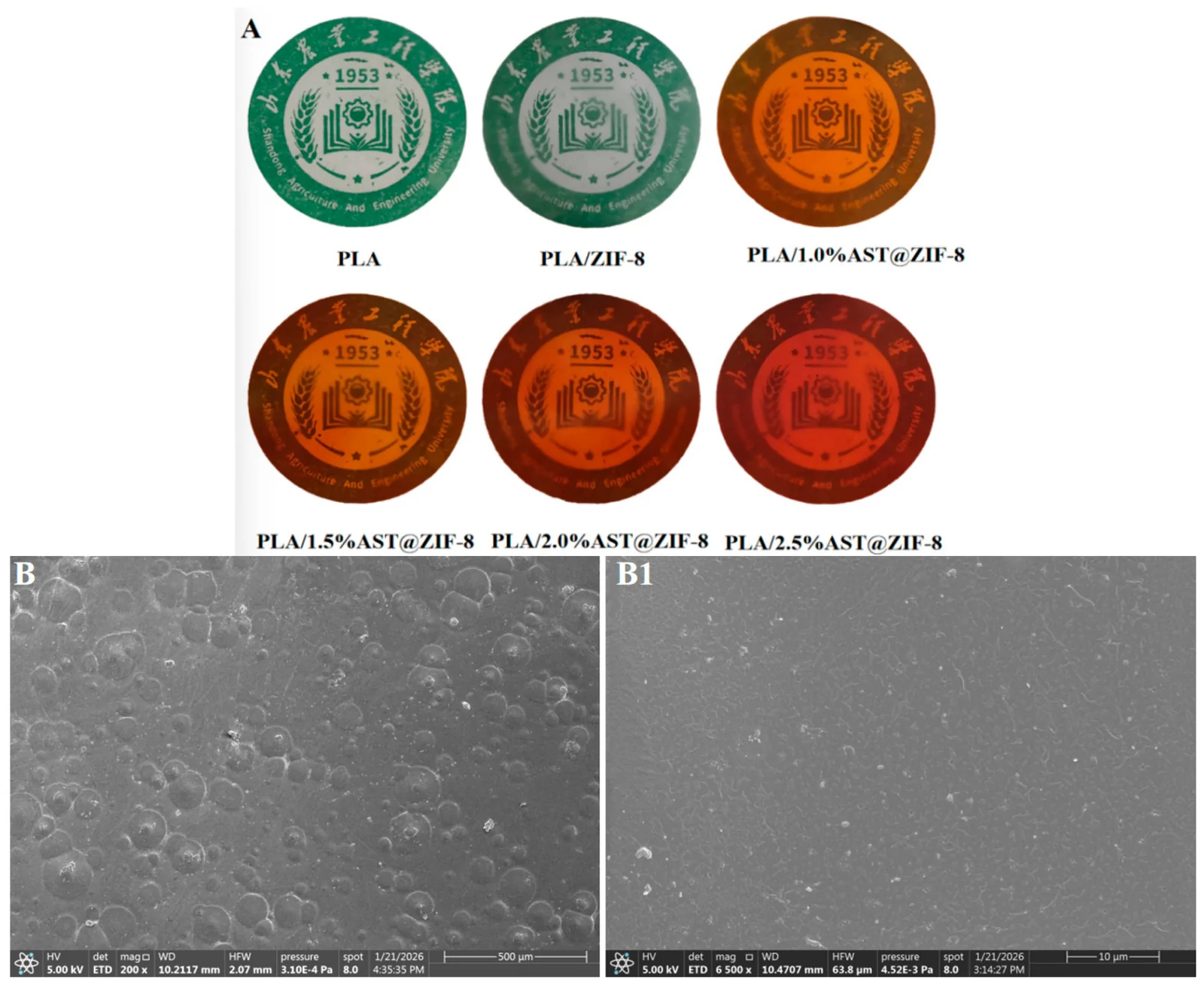

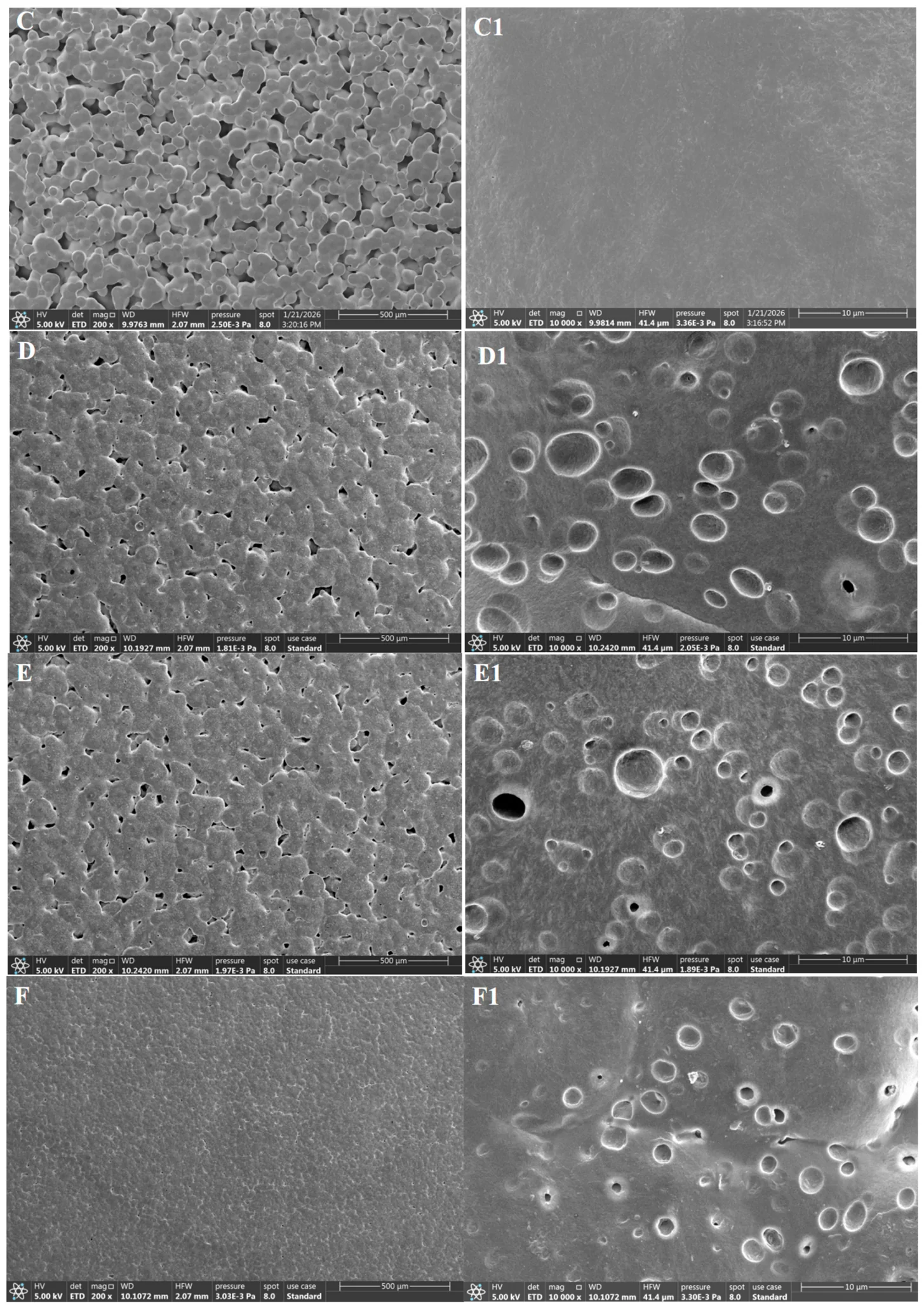

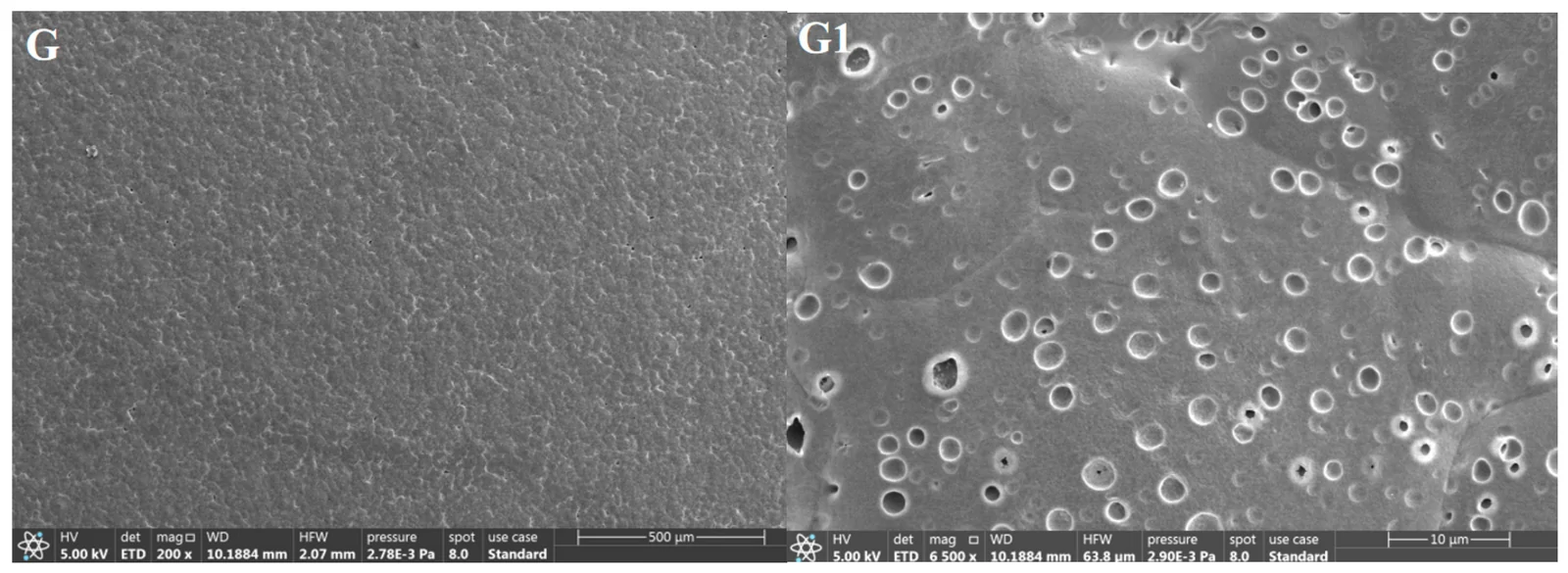
(**A**) Digital photos of different film samples; FE-SEM microstructures of (**B**,**B1**) PLA, (**C**,**C1**) PLA/ZIF-8, (**D**,**D1**) PLA/1.0%AST@ZIF-8, (**E**,**E1**) PLA/1.5%AST@ZIF-8, (**F**,**F1**) PLA/2.0%AST@ZIF-8, (**G**,**G1**) PLA/2.5%AST@ZIF-8.

Figure 2A presents optical micrographs of the prepared PLA, PLA/ZIF-8 and PLA/AST@ZIF-8 films. As observed, the absence of visible ZIF-8 or AST aggregates within the films indicates their excellent dispersibility in the PLA matrix. During film preparation, vacuum drying was found to cause excessively rapid solvent evaporation, leading to significant bubbling, surface porosity, and potential whitening or crystallization, which compromised film quality. In contrast, utilizing a constant temperature and humidity incubator (35 °C, 80% relative humidity (RH)) yielded films with a uniform appearance and good transparency. The incorporation of ZIF-8 into PLA resulted in a slight haze, while the subsequent addition and increasing concentration of AST caused the film color to transition from colorless to AST’s characteristic red hue.

Figure 2B–G displays the FE-SEM micrographs of the PLA, PLA/ZIF-8, and PLA/AST@ZIF-8 films. Figure 2B,B1 present the representative surface morphology of crystallized PLA, which exhibits a relatively smooth surface accompanied by irregular spherical protrusions within crystalline domains. Distinct morphological changes can be seen for the PLA/ZIF-8 film (Figure 2C,C1). Compared with neat PLA, its surface is dominated by block-shaped features together with honeycomb-like porous architecture composed of abundant surface pits. The introduction of ZIF-8 markedly modifies the surface architecture of PLA and greatly alters its crystallization behavior [41]. Neat PLA is incapable of forming such well-defined honeycomb pores. However, under controlled temperature-humidity conditions (35 °C, 80% RH), incorporated ZIF-8 nanoparticles trigger heterogeneous solvent-water evaporation during PLA crystallization. This effect drives the development of ordered honeycomb porous architecture populated by abundant surface pits and gives rise to increased surface roughness. Such pit-rich rough topography increases the specific surface area of the PLA matrix, which facilitates higher loading of bioactive guest molecules [42]. In addition, negligible particle agglomeration observed in PLA/ZIF-8 film confirms the good dispersion of ZIF-8 nanoparticles throughout the PLA matrix.

With increasing AST loading from 1.0% to 1.5%, the PLA/AST@ZIF-8 films displayed polymer crystalline features analogous to those of PLA/ZIF-8, accompanied by a more compact surface morphology. As shown in Figure 2D,E, small-sized honeycomb-like pores with diameters ranging from 1.5 to 4.5 µm were generated on the film surface (Figure 2D1,E1). Upon further raising AST content to 2.0–2.5%, the surfaces of PLA/AST@ZIF-8 (Figure 2F,G) became denser. Large irregular interfacial voids vanished, while the honeycomb-structured pores became more homogeneous, with a confined diameter of approximately 2.5 µm (Figure 2F1,G1). The enhanced compactness of PLA/AST@ZIF-8 composite films can be ascribed to the homogeneous dispersion of AST@ZIF-8 nanoparticles within the PLA matrix. Meanwhile, strengthened interfacial interactions between ZIF-8-encapsulated AST molecules and PLA chains promote the regular arrangement of PLA crystals. The improved homogeneity of honeycomb pores originates from abundant and well-distributed nucleation sites provided by AST@ZIF-8, as well as the stable immobilization of AST within ZIF-8 cavities. Collectively, the favorable particle dispersibility and matrix-filler compatibility contribute to the optimized surface microstructure of the resultant composite films.

### 3.2. FT-IR, Raman, XRD and TGA of the PLA, PLA/ZIF-8 and PLA/AST@ZIF-8 Composite Films

**Figure 3:**
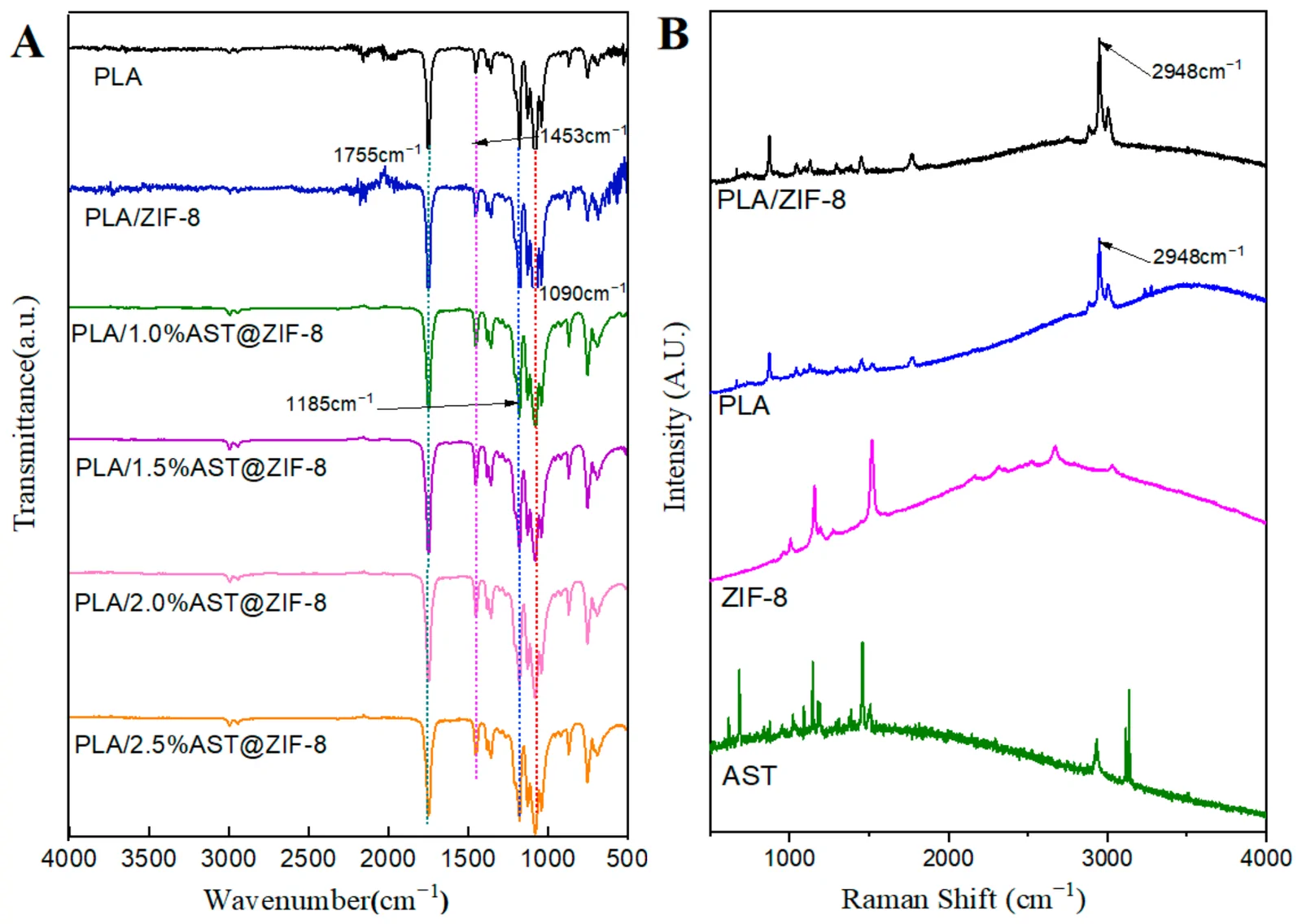

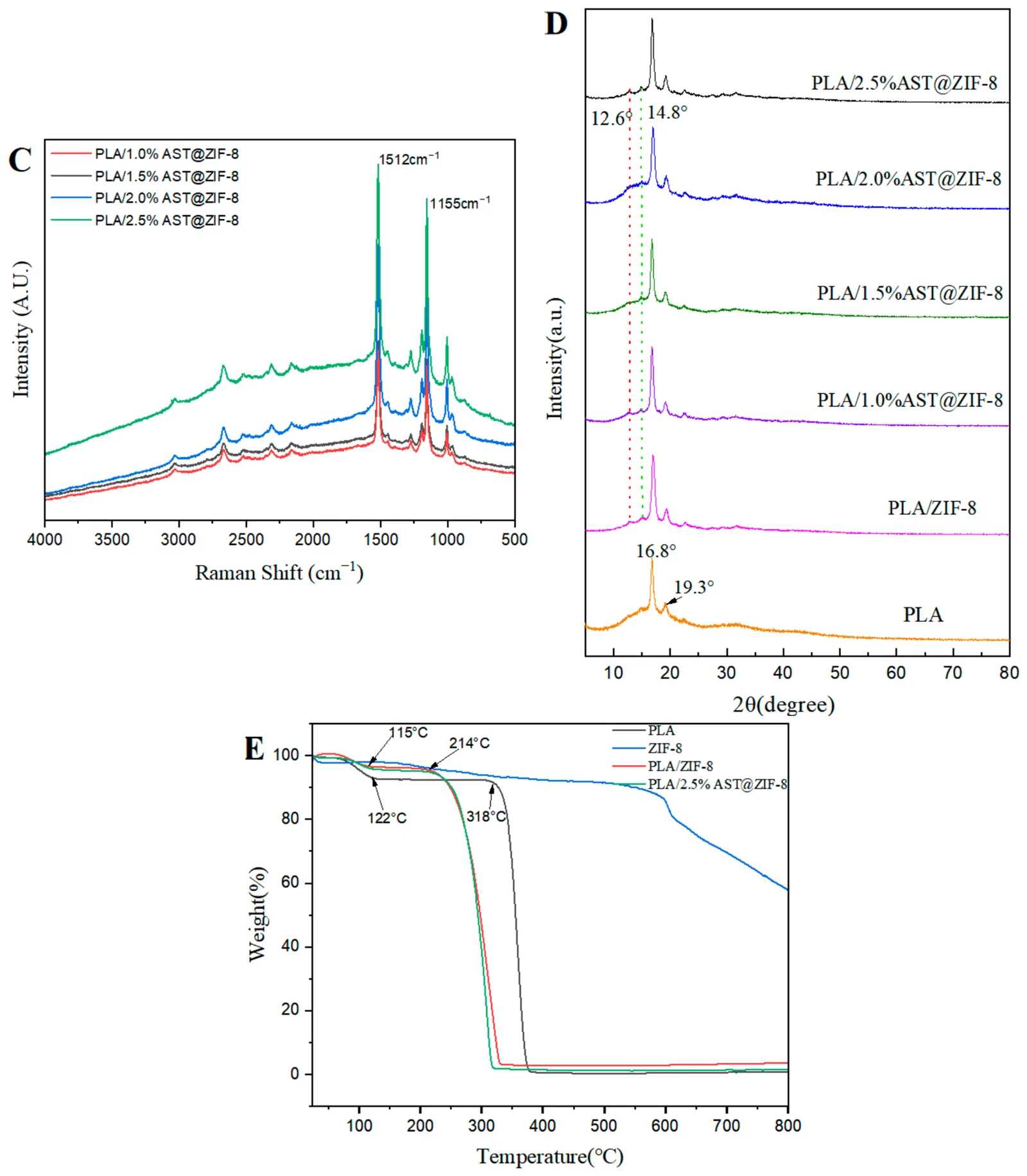
(**A**) FTIR spectra of the PLA, PLA/ZIF-8 and PLA/AST@ZIF-8 composite films; (**B**) Raman spectra of the PLA, ZIF-8, AST and PLA/ZIF-8; (**C**) Raman spectra of the PLA/AST@ZIF-8 composite films; (**D**) XRD of the PLA, PLA/ZIF-8 and PLA/AST@ZIF-8 composite films; (**E**) TGA curves of the PLA, ZIF-8, PLA/ZIF-8 and PLA/2.5%AST@ZIF-8 composite films under N_2_ atmosphere.

Figure 3A presents the FTIR spectra of neat PLA, PLA/ZIF-8, and PLA/AST@ZIF-8 composite films. For neat PLA matrix, the characteristic absorption bands located at 1755 cm^−1^ and 1453 cm^−1^ correspond to the C=O stretching vibration and C-H bending vibration, respectively [43]. The adsorption peaks at 1090 cm^−1^ and 1186 cm^−1^ are assigned to the symmetric and asymmetric stretching vibrations of the C-O-C ether bonds in the PLA molecular chains, respectively. After the incorporation of ZIF-8 nanoparticles, no significant shift or disappearance of the above typical PLA characteristic peaks was observed, demonstrating that the introduction of ZIF-8 does not alter the intrinsic chemical structure of the PLA backbone. Notably, distinct AST characteristic peaks were not detected in the FTIR spectra of PLA/AST@ZIF-8 composites, which can be attributed to the low surface content and highly encapsulated state of AST molecules within the ZIF-8 framework. Given the limited sensitivity of FTIR for trace bioactive components, Raman spectroscopy was further employed to elucidate the interfacial interaction and molecular composition of the composite films. Figure 3B,C display the Raman spectra of the prepared films, AST, and ZIF-8. In contrast to the PLA/ZIF-8 group, the composite films exhibited distinct AST characteristic Raman signals, whose peak intensity varied regularly with the adjustment of AST loading proportion. Moreover, the absence of prominent ZIF-8 characteristic peaks in the PLA/AST@ZIF-8 composites indicates strong interfacial binding and favorable compatibility between ZIF-8 nanoparticles and the PLA matrix, which further verifies the successful fabrication of integrated composite films rather than simple physical blending.

The XRD patterns of the fabricated samples are illustrated in Figure 3D. For neat PLA films, the prominent diffraction peaks located at *2θ* = 16.8° and 19.3° are indexed to the (110), (200), and (203) crystal planes of PLA, respectively [44]. With the gradual increase in AST loading, the diffraction intensities of the two characteristic PLA peaks are remarkably enhanced, demonstrating an improved crystallinity of the resultant composite films. Typical characteristic diffraction peaks of ZIF-8 at 12.6° and 14.8° were clearly detected in both PLA/ZIF-8 and PLA/AST@ZIF-8 composites. Notably, the characteristic ZIF-8 diffraction peak at 7.1° was absent in all composite films [19]. This phenomenon indicates that ZIF-8 nanoparticles are well encapsulated inside the PLA matrix without surface exposure, revealing the favorable composite interfacial structure, which is highly consistent with the aforementioned FE-SEM morphological observations.

The thermal stability of neat PLA, PLA/ZIF-8, and PLA/2.5%AST@ZIF-8 composite films was systematically investigated via thermogravimetric analysis (TGA), and the corresponding thermograms are presented in Figure 3E. The neat PLA film exhibited an initial thermal decomposition at 122 °C with a residual weight retention of 92.9%, followed by a major mass loss stage starting at 318 °C, which corresponds to the intensive thermal degradation of PLA molecular chains. Neat ZIF-8 maintained excellent thermal stability throughout the entire heating procedure, which is in good agreement with previously reported literature [45]. For the PLA/ZIF-8 composite film, a slight preliminary weight loss was observed at 115 °C with a residual weight of 96.7%, and the dominant thermal decomposition commenced at 214 °C. In addition, the PLA/2.5%AST@ZIF-8 composite films displayed a comparable thermal degradation behavior and thermal stability to the PLA/ZIF-8 counterpart, indicating that the introduction of AST encapsulated by ZIF-8 barely compromises the thermal performance of the PLA composite system. Combined with SEM observations, the formed honeycomb porous microstructure of PLA/2.5%AST@ZIF-8 films facilitates the dispersion of fillers within polymer matrix, which further accounts for the improved thermal stability shown in the initial heating stage. Further TGA demonstrated that the incorporation of ZIF-8 effectively enhanced the thermal stability of the PLA/ZIF-8 composite. In comparison, the PLA/2.5%AST@ZIF-8 composites exhibited negligible improvement in thermal stability relative to neat PLA. This distinct thermal characteristic is primarily attributed to the successful embedding of AST molecules within the ZIF-8 framework, which weakens the thermal protection effect of pristine ZIF-8. Importantly, the retained moderate thermal stability confers the composite films with favorable biodegradability, offering great potential for sustainable and eco-friendly food packaging systems.

### 3.3. Surface Hydrophobicity and OP of Different Films

**Figure 4:**
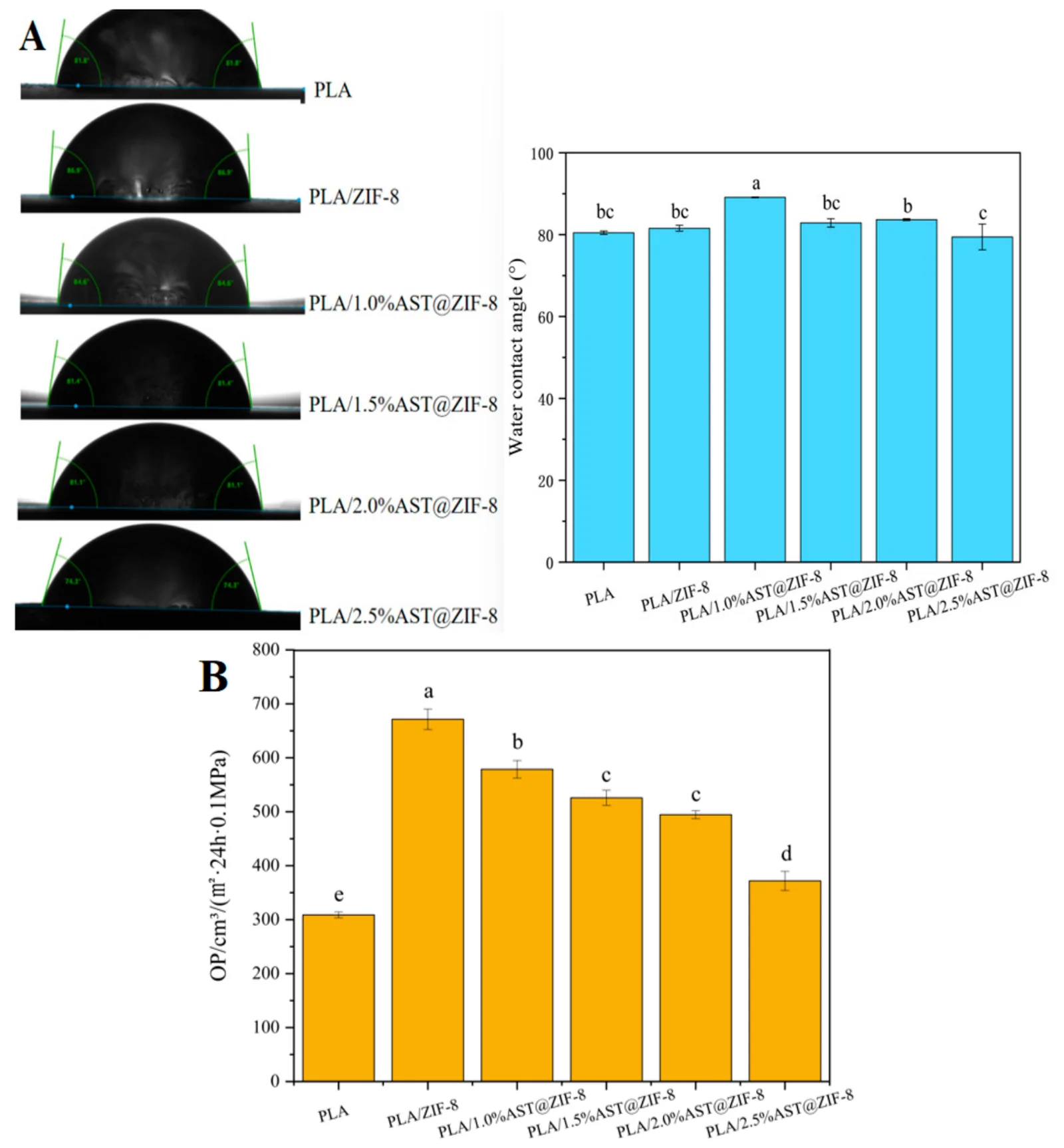
(**A**) Water contact angle and (**B**) OP of the PLA, PLA/ZIF-8 and PLA/AST@ZIF-8 composite films. Data were presented as M ± SD (*n* = 3), different letters showed significantly different (*p* < 0.05).

Hydrophobicity and OP are critical performance indicators for food packaging materials, which serve as essential barriers against food oxidation, deterioration, and spoilage to preserve product quality and prolong shelf life. Effective isolation of external oxygen molecules can efficiently inhibit the oxidative degradation and spoilage of food products [46]. The surface hydrophobicity of composite films was evaluated via water contact angle (CA) measurement. As depicted in Figure 4A, the incorporation of AST@ZIF-8 nanoparticles induced no remarkable variation in the CA values of PLA composite films. This result demonstrates that the introduction of AST@ZIF-8 has a negligible effect on the surface wettability of the fabricated films.

As shown in Figure 4B, the OP value of PLA/ZIF-8 film is significantly higher than that of PLA film, indicating poor adhesion between the ZIF-8 nanoparticles and PLA, resulting in increased OP of the composite film. With the addition of AST@ZIF-8 nanoparticles, the OP value of PLA/AST@ZIF-8 composite film shows a decreasing trend with the increase in AST content. The OP value of PLA/2.5%AST@ZIF-8 film is basically consistent with that of PLA, indicating that AST@ZIF-8 nanoparticles have strong interfacial interactions (such as hydrogen bonds and van der Waals forces) with PLA molecular chains in the composite film, resulting in a more regular arrangement of PLA molecular chains around the nanoparticles and reducing the free volume of non-crystalline regions [47], which is consistent with the above FE-SEM results.

### 3.4. Antibacterial Activities of the Composite Films

**Figure 5:**
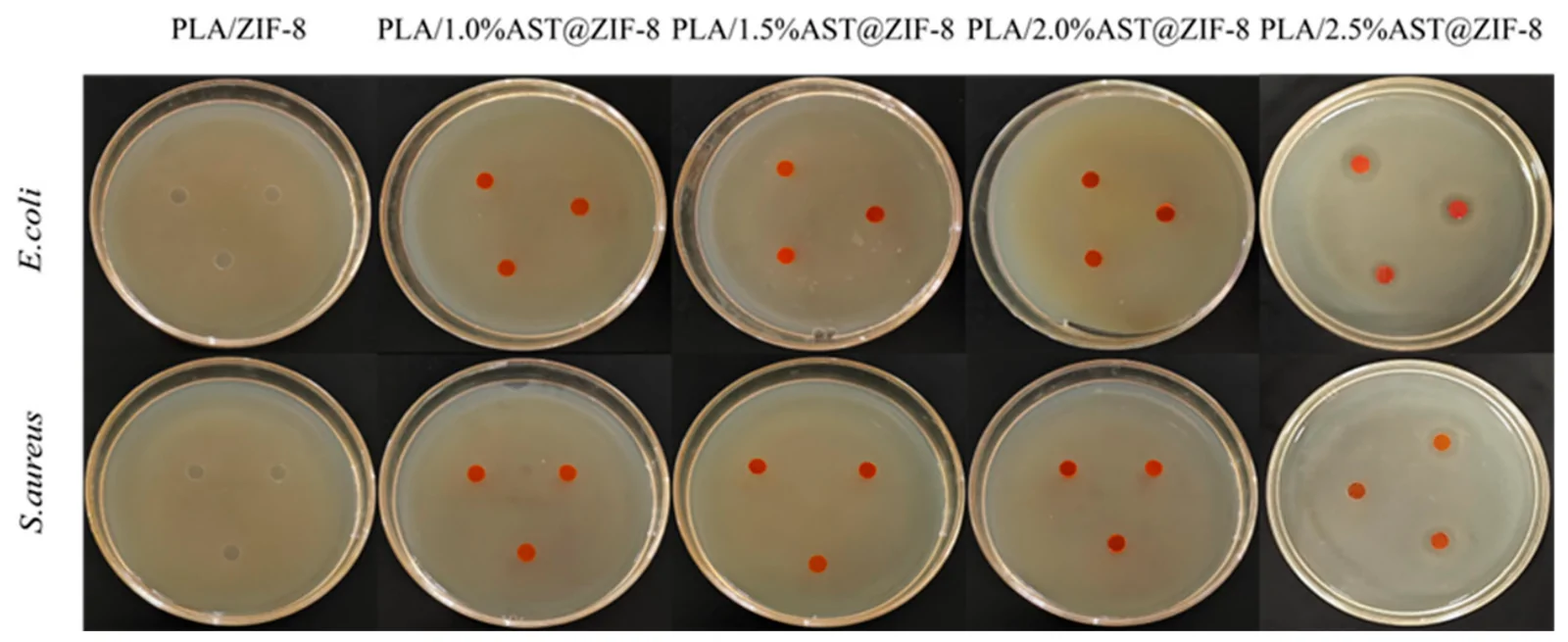
The inhibition zone of composite films for *E. coli* and *S. aureus*.

The inhibitory activity of composite films was determined against *E. coli* and *S. aureus*. As shown in Figure 5, for PLA/ZIF-8 films, no obvious inhibition zone was observed, implying that the addition of ZIF-8 did not alter the antibacterial properties of PLA. Some research has shown that the release of Zn^2+^ from ZIF-8 exhibits certain antibacterial properties [47]; thus, it can be inferred that ZIF-8 did not migrate out of the PLA film in PLA/ZIF-8. At the same time, it was also found that with the increase in AST content in PLA/AST@ZIF-8 films (AST content *w*/*w*, from 1.0% to 2.5%), the inhibition zone gradually increased. This indicates that the content of AST in the film above 2.0% will produce weak antibacterial properties. This finding aligned with previous studies indicating that AST had a moderate or weak inhibitory effect on Gram-positive bacteria *S. aureus* and Gram-negative bacteria *E. coli* [48]. As proposed by Karpiński et al., AST’s antibacterial mechanisms resemble other carotenoids and terpenoids [49]. They increase bacterial membrane permeability and regulate efflux pumps, leading to cytoplasmic leakage accumulating toxic compounds in bacterial cells to disrupt their activation [50,51]. As an antioxidant, AST inhibits bacterial oxygen uptake and induces reactive oxygen species (ROS) accumulation, damaging bacterial structures [49,52]. AST not only exerts bacteriostatic effects but also functions as an antioxidant, which further improves the preservation performance of composite packaging films.

### 3.5. Food Packaging Applications for Beef Tenderloin

#### 3.5.1. Measurement of Water Loss in Beef Tenderloin

**Figure 6:**
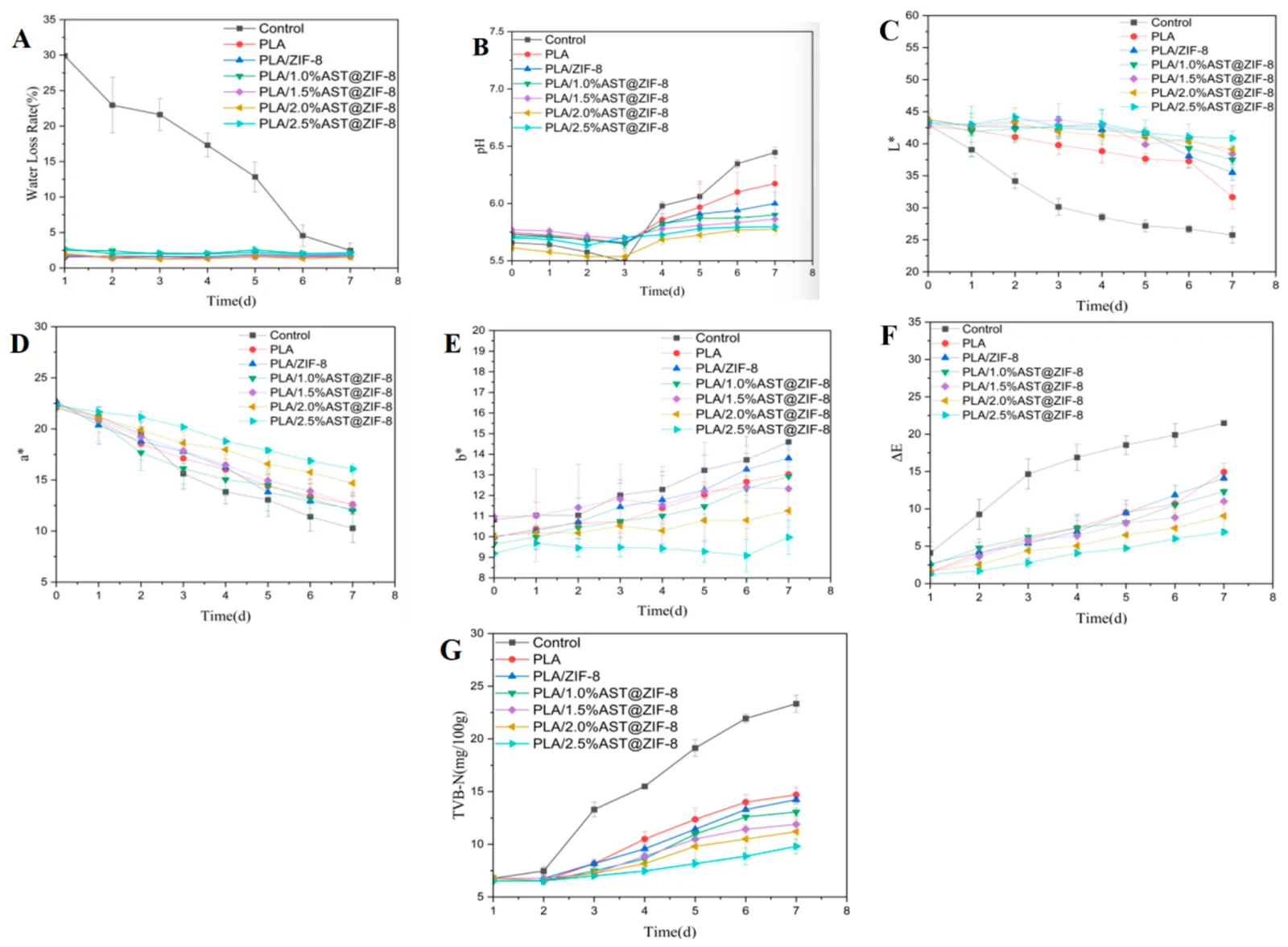
Changes in (**A**) water loss, (**B**) pH, (**C**) L*, (**D**) a*, (**E**) b*, (**F**) *ΔE*, and (**G**) TVB-N of the beef sample packaged with the as-prepared composite films at the different storage time and during 4 ± 1 °C.

Water loss in beef tenderloin during refrigerated storage primarily originates from water evaporation, which inevitably causes the loss of water-soluble vitamins, sarcoplasmic proteins, and other nutritional components [53]. The water loss rates of beef samples with different film treatments during storage are presented in Figure 6A. The blank group exhibited a significantly higher water loss rate than all PLA/AST@ZIF-8 treatment groups and showed a declining trend throughout storage. This variation is mainly attributed to the rapid evaporation of free water in beef tissues at the early storage stage, while tightly bound water remains relatively stable in the later stage. Notably, all PLA/AST@ZIF-8 composite film groups (AST loading of 1.0%, 1.5%, 2.0%, and 2.5%) maintained a water loss rate below 3.0% during 7 days of refrigeration, among which the PLA/2.5% AST@ZIF-8 group achieved the lowest water loss. Combined with the FE-SEM microscopic morphology results, the enhanced water retention capability is closely correlated with the AST-dependent microstructure evolution of composite films. Specifically, at low AST loading (1.0–1.5%), PLA/AST@ZIF-8 films retained crystalline characteristics similar to pure PLA/ZIF-8, with uniform small-sized honeycomb pores (1.5–4.5 µm) formed on the film surface. With further elevation of AST content to 2.0–2.5%, the film microstructure was significantly optimized: large irregular interfacial voids disappeared, and the honeycomb pore structure became more homogeneous, accompanied by a markedly denser and more compact film surface. This progressive structural optimization effectively restricts capillary water migration within the film matrix [54]. The optimized dense barrier structure greatly suppresses internal water evaporation from beef tenderloin, alleviates nutritional component loss, and effectively maintains the original juiciness of meat samples during refrigerated storage.

Furthermore, the water loss rate exhibited a distinct AST concentration-dependent tendency. With the increase in AST loading from 1.0% to 2.5%, the water loss of beef gradually decreased, demonstrating a synergistic water-retention effect between AST bioactive components and the hierarchical composite film structure.

#### 3.5.2. Measurement of pH in Beef Tenderloin

The pH changes in beef tenderloin during the 7-day refrigeration period are shown in Figure 6B. The pH values of all samples showed a trend of first decreasing and then increasing, reaching the lowest value on the third day of storage. The initial decrease in pH value is mainly due to the production of lactic acid by glycogen fermentation after beef slaughter, which leads to a decrease in tissue pH. As the storage time increases, the pH value significantly increases, which is due to the decomposition of proteins and amino acids by spoilage microorganisms (such as *E. coli*, *S. aureus*, etc.), resulting in the accumulation of alkaline metabolites such as amines and ammonia [55]. Compared with the blank control group and the pure PLA film group, the pH increase rate of the PLA/AST@ZIF-8 composite film treatment group significantly slowed down during the 3–7 day storage period. Throughout the refrigeration process, the pH value of the PLA/AST@ZIF-8 group beef remained lower than that of the pure PLA group and the blank control group. Among them, the PLA/2.0%AST@ZIF-8 and PLA/2.5%AST@ZIF-8 composite films maintained a pH value below 5.75 during the 7-day storage period. The above results indicate that the PLA/AST@ZIF-8 composite membrane can effectively inhibit the metabolic activity of spoilage microorganisms. At the molecular level, AST molecules are firmly immobilized within ZIF-8 pores via strong intermolecular forces including hydrogen bonding and electrostatic interactions. This confinement protects AST against structural degradation and retains its intrinsic biological activity. Notably, synergistic biomolecular cooperation between AST and ZIF-8 enhances overall preservation performance. The fabricated PLA/AST@ZIF-8 composite films possess a distinctive honeycomb-structured porous architecture, which enlarges the effective specific surface area for AST release, facilitates active-site exposure, and improves antioxidant efficiency. Meanwhile, the pore confinement of ZIF-8 facilitates sustained release of encapsulated AST, while bioactive AST endows the inert PLA matrix with prominent antioxidant and bacteriostatic capacities. This combined structural and biomolecular synergy suppresses the accumulation of bacterial alkaline metabolites, delays pH rise in meat samples, and establishes a highly efficient biomolecular preservation system for chilled meat.

#### 3.5.3. Measurement of *ΔE* in Beef Tenderloin

**Figure 7:**
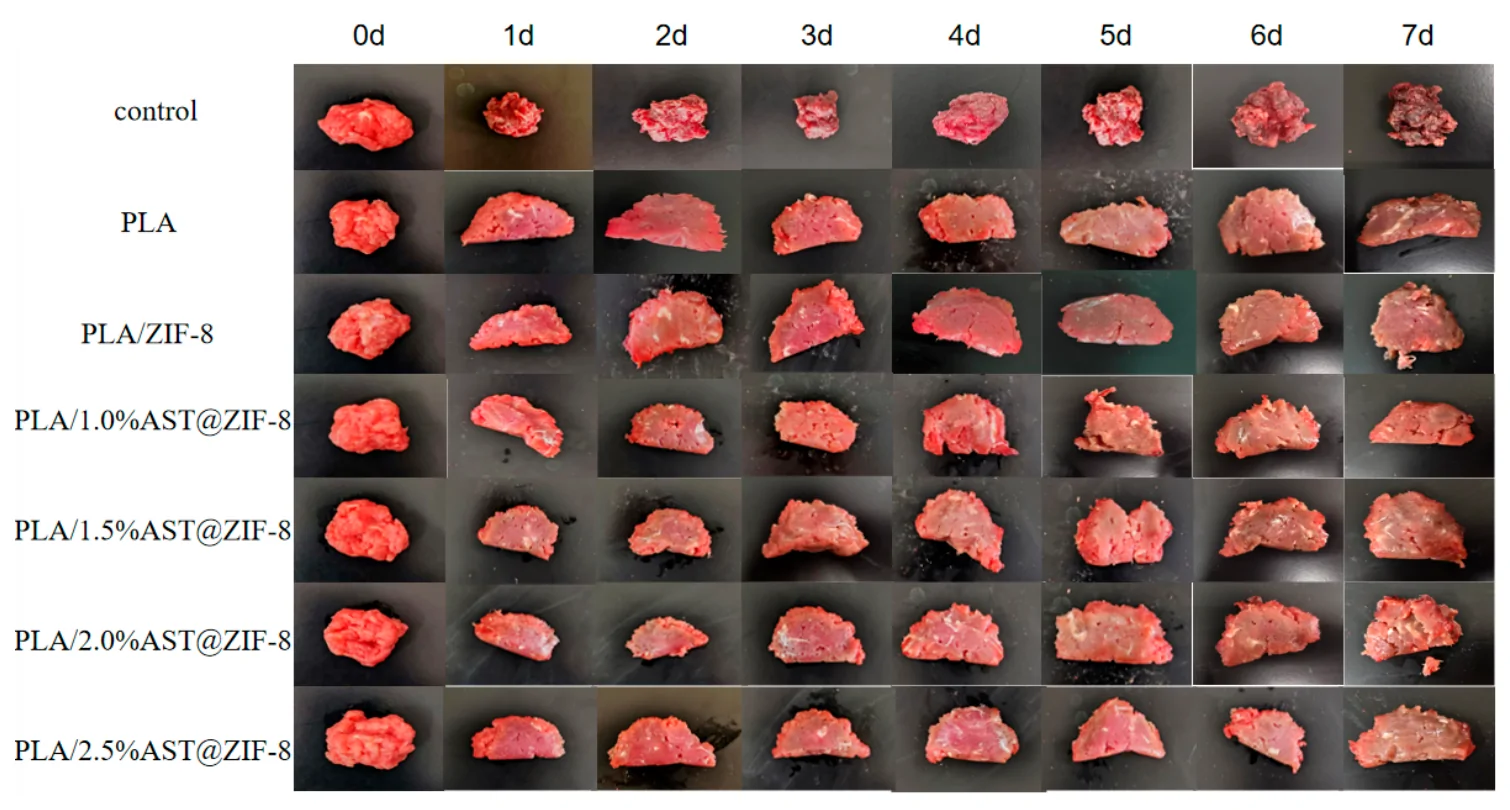
Appearance of packaged beef samples during 4 ± 1 °C storage.

Color is a key sensory indicator that affects consumer purchasing decisions, and the color stability of beef is closely related to the oxidation status of myoglobin in muscle tissue [56]. The changes in color parameters (L*, a*, b*, *ΔE*) of beef samples during refrigeration are shown in Figure 6C. The L* values of all groups gradually decreased during storage, due to myoglobin oxidation and accumulation of microbial metabolites, resulting in darkening of meat quality. However, the rate of decrease in L* value of the PLA/AST@ZIF-8 composite film treatment group slowed down with increasing AST concentration. On the 7d of storage, the L* value of the PLA/2.5% AST@ZIF-8 group was significantly higher than other groups, still maintaining above the acceptable threshold (31.4), while the L* value of the blank control group decreased to below 25.0, indicating severe discoloration. The a* value (representing redness) of beef is directly related to the content of oxygenated myoglobin and serves as a critical indicator for evaluating meat freshness. As illustrated in Figure 6D, all groups exhibited a gradual decline in a* values during refrigerated storage. Nevertheless, the PLA/AST@ZIF-8-treated beef consistently maintained higher a* values than the blank and neat PLA groups, presenting an AST concentration-dependent anti-browning effect. Higher AST loading effectively retarded the reduction in redness. On day 7, the PLA/2.5% AST@ZIF-8 group still retained an a* value above the commercially acceptable threshold (14.5), whereas the blank control group decreased below 10.0, indicating severe meat browning and oxidative deterioration. This is because AST has strong antioxidant activity, and its phenolic hydroxyl group can clear free radicals generated during storage, inhibit the oxidation of Fe^2+^ in myoglobin to Fe^3+^, reduce the production of metmyoglobin, and maintain the bright red color of beef. The b* values of all treatment groups (Figure 6E) gradually increased during the later stage of storage (5–7d), mainly due to the combination of hydrogen sulfide produced by microbial protein decomposition with hemoglobin, forming a yellow green complex from the H_2_S binding to hemoglobin [25]. However, the increase in b* value in the PLA/AST@ZIF-8 group was significantly lower than that in the control group, especially in the 2.5% AST group, indicating that the composite film can inhibit microbial metabolism and reduce hydrogen sulfide production. The total color difference *ΔE* (Figure 6F) characterizes the overall color variation in beef samples relative to their initial status. As observed from the curves, the blank control group exhibited a rapid rise in *ΔE* values throughout storage. By contrast, the PLA/2.5% AST@ZIF-8 group maintained *ΔE* below 6 even on day 7, confirming favorable color-retention capacity of the composite film. Consistent with quantitative color analysis, visual photographs of beef over 7 days further verify the distinct preservation performance of different films (Figure 7). The untreated control deteriorated rapidly with severe discoloration starting at day 1. The pristine PLA slightly delayed oxidation but failed to sustain beef redness, and PLA/ZIF-8 achieved only moderate preservation. PLA/AST@ZIF-8 composites delivered prominent fresh-keeping capacity. Although the PLA/2.0% AST@ZIF-8 sample efficiently retained red color and inhibited browning, increasing AST loading to 2.5% yielded extra improvements. Overall, these results confirm the synergistic effect of PLA/AST@ZIF-8 composite films optimizing the barrier structure, reducing the OP of film, thereby slowing down the oxidation rate of oxygenated myoglobin and enhancing color stability.

#### 3.5.4. Measurement of TVB-N in Beef Tenderloin

TVB-N serves as a critical biomarker for evaluating meat freshness, which quantifies the extent of microbial-mediated protein catabolism that generates alkaline nitrogenous metabolites such as ammonia, trimethylamine, and dimethylamine [57]. In accordance with Chinese national standard GB/T 9959.2-2008 [58], beef is considered spoiled and unfit for human consumption once TVB-N content exceeds 15 mg/100 g. Figure 6G illustrates the dynamic evolution of TVB-N in beef samples subjected to different packaging treatments over refrigerated storage. TVB-N concentrations increased markedly across all experimental groups with prolonged storage. The blank control exhibited the most rapid TVB-N accumulation, surpassing the regulatory threshold of 15 mg/100 g on day 4 and reaching around 20 mg/100 g by day 7, indicative of substantial microbial spoilage. The neat PLA group also approached this critical limit at the end of storage. In contrast, packaging with PLA/AST@ZIF-8 composite films greatly suppressed TVB-N build-up. Even on day 7, samples wrapped with 1.5% and 2.0% AST-loaded composite films maintained TVB-N below 13 mg/100 g, confirming the effective preservation of meat freshness owing to the antimicrobial and barrier functions of the composite films. The PLA/2.5%AST@ZIF-8 group had the lowest TVB-N content on the seventh day, still remained within the acceptable threshold for fresh beef. This result confirms that the PLA/AST@ZIF-8 composite films have excellent antibacterial and oxygen barrier synergistic effects. The ZIF-8 component provides a physical barrier to inhibit microbial invasion, while AST further inhibits the growth and reproduction of spoilage bacteria, thereby reducing protein degradation and TVB-N accumulation. The meat preservation performance of the as-prepared AST@ZIF-8-based PLA active material is comparable to that of several reported active biodegradable packaging materials, including PEO/casein/thymol/β-cyclodextrin, carboxymethyl-starch/shallot wastes, chitosan/PEO/pomegranate peel extract, and chitosan nanofibers [59].

### 3.6. OM and Zn^2+^ Ion Migration Test

OM- and Zn^2+^-specific migration of the as-prepared PLA/AST@ZIF-8 composite films were evaluated under 7-day contact conditions following GB 5009.156-2016. Four aqueous ethanol solutions (10%, 20%, 50%, and 65%) were employed as food simulants to quantify OM. A 4% acetic acid solution, the standard acidic food simulant, was used to determine Zn^2+^-specific migration. Migration measurements demonstrated that both overall-migration and Zn^2+^-specific-migration values for PLA/AST@ZIF-8 films (AST loading: 1.0–2.5%) were below 10 mg/dm^2^ across all tested simulants. All obtained migration levels satisfy the regulatory limits specified in EU Regulation No. 10/2011 [60]. These findings confirm that the developed composite films fulfil safety prerequisites and are suitable for food-contact packaging applications.

## 4. Conclusions

In this work, a novel honeycomb-structured PLA/AST@ZIF-8 composite film was successfully fabricated via microstructure regulation and bioactive component encapsulation, with PLA serving as the polymer matrix and assisted by constant temperature and humidity optimization. The ZIF-8 scaffold not only enables stable immobilization and sustained loading of AST molecules, but also constructs a uniform honeycomb porous microstructure through its inherent porous characteristics synergized with environmental regulation. This unique structural feature provides a favorable structural basis for the optimized comprehensive performance of composite films. Performance characterization verified that the optimized PLA/AST@ZIF-8 films possess prominent antibacterial and antioxidant capacities. The composite barrier system can effectively inhibit microbial proliferation and retard the oxidative deterioration of meat samples, thereby significantly improving food preservation efficiency. Moreover, migration safety tests demonstrated that the as-prepared composite films fully comply with international food-contact safety standards without potential safety hazards. Benefiting from the synergistic effect of component optimization and structural design, the PLA/AST@ZIF-8 composite films integrate excellent antimicrobial activity, antioxidant performance, and reliable biosafety. Accordingly, this innovative composite material presents promising application potential in high-efficiency food preservation and green packaging fields, and provides a feasible strategy for the design and development of next-generation, high-performance, and eco-friendly active packaging films.

## Data Availability

The original contributions presented in the study are included in the article, further inquiries can be directed to the corresponding author.
